# Volumetric changes in the maxillary sinus following orthodontic disimpaction of unilaterally impacted maxillary canines: a prospective CBCT-based split-mouth study

**DOI:** 10.1590/2177-6709.30.6.e252554.oar

**Published:** 2026-01-23

**Authors:** Neha KUMARI, Chaitra Santoshkumar MASTUD, Swati V. PISSAY, Asmita KHARCHE, Sonali DESHMUKH, Jayesh RAHALKAR

**Affiliations:** 1 Private practice, Orthodontic Clinics, F1 SriKrishna Regency A block (Jharkhand, India). Jharkhand India; 2 Dr. D.Y. Patil Vidyapeeth University Pimpri-Pune, Dr. D.Y. Patil Dental College and Hospital, Department of Orthodontics and Dentofacial Orthopedics (Pimpri Chinchwad, India). Dr. D.Y. Patil Vidyapeeth University Pimpri-Pune Dr. D.Y. Patil Dental College and Hospital Department of Orthodontics and Dentofacial Orthopedics Pimpri Chinchwad India

**Keywords:** Maxillary canine, Impaction, Unilateral, Maxillary sinus, Volume, Canino superior, Impacção, Unilateral, Seio maxilar, Volume

## Abstract

**Introduction:**

The relationship between orthodontic disimpaction of unilaterally impacted maxillary canines (MCs) and volumetric changes in the maxillary sinus (MS) remains underexplored.

**Objective:**

This study aimed to evaluate MS volume alterations following orthodontic traction of impacted MCs, using cone-beam computed tomography (CBCT). Additionally, it examined differences based on the impaction site (buccal vs. palatal) and sex.

**Material and Methods:**

A prospective split-mouth study was conducted on patients with unilaterally impacted MCs. CBCT scans were taken before and after orthodontic disimpaction. MS volume was measured using three-dimensional reconstruction software. Comparisons were made between the impacted and non-impacted sides, buccal and palatal impactions, and male and female patients. Statistical analysis included paired t-tests and analysis of variance (ANOVA) for group comparisons. A multifactorial regression analysis was performed to identify predictor variables influencing MS volume normalization.

**Results:**

A significant increase in MS volume was observed on the previously impacted side, following orthodontic disimpaction (p < 0.05). Buccally impacted MCs showed greater pretreatment sinus volume, compared to palatally impacted MCs. Additionally, statistically significant sex-based differences were not noted, in the relative volumetric change between sexes (p > 0.05). It was indicated by our study that younger age, palatally impacted MCs, shorter treatment duration, increased distance of the root tip of impacted MC from MS floor, and pretreatment MS volume differences were significant predictors of MS volume normalization.

**Conclusion:**

Orthodontic disimpaction of unilaterally impacted MCs influenced MS volume, with greater mean change of MS volume in palatal impactions.

## RESUMO

**Introdução:**

A relação entre a desimpacção ortodôntica de caninos superiores (CSs) impactados unilateralmente e as alterações volumétricas no seio maxilar (SM) permanece pouco explorada.

**Objetivo:**

Este estudo teve como objetivo avaliar as alterações volumétricas do SM após a tração ortodôntica de CSs impactados, utilizando tomografia computadorizada de feixe cônico (TCFC). Adicionalmente, examinou as diferenças com base no local da impacção (vestibular vs. palatina) e no sexo.

**Material e Métodos:**

Foi realizado um estudo prospectivo de boca dividida em pacientes com CSs impactados unilateralmente. As TCFCs foram obtidas antes e depois da desimpacção ortodôntica. O volume do SM foi mensurado utilizando um *software* de reconstrução tridimensional. Foram feitas comparações entre os lados impactado e não impactado, entre impacções vestibulares e palatinas, e entre pacientes dos sexos masculino e feminino. A análise estatística incluiu testes t pareados e análise de variância (ANOVA) para comparações entre os grupos. Uma análise de regressão multifatorial foi realizada para identificar variáveis preditoras que influenciam a normalização do volume do SM.

**Resultados:**

Observou-se um aumento significativo no volume do SM no lado previamente impactado, após a desimpacção ortodôntica (p < 0,05). Os CSs impactados por vestibular apresentaram maior volume do SM antes do tratamento, em comparação com os CSs impactados por palatina. Além disso, não foram observadas diferenças estatisticamente significativas entre os sexos na variação volumétrica relativa (p > 0,05). O presente estudo indicou que idade mais jovem, CSs impactados por palatina, menor duração do tratamento, maior distância da ponta da raiz do CS impactado em relação ao assoalho do SM e diferenças no volume do SM antes do tratamento foram preditores significativos da normalização do volume do SM.

**Conclusão:**

A desimpacção ortodôntica de CSs impactados unilateralmente influenciou o volume do SM, com maior variação média do volume do SM em impacções palatinas.

## INTRODUCTION

The maxillary sinus (MS), the largest paranasal sinus, is pyramidal in shape, with its base in the lateral nasal wall and apex in the zygomatic process. Its volume increases from 6-8 cm³ at birth to 8.6-24.9 cm³ by 12-15 years of age.^1^ Maxillary canines (MCs), erupting between 11-13 years, may deviate from their path, with 67% impacted palatally and 33% labially, affecting 1.38% of Indian population.^2^^,^^3^ The roots of impacted MCs are found to be closer to MS. Therefore, the evaluation of alterations in MS volume subsequent to the orthodontic disimpaction of unilaterally displaced MCs, regardless of whether they are buccally or palatally impacted, constitutes a subject of considerable clinical importance. 

Research using cone-beam computed tomography (CBCT) has demonstrated that the presence of an impacted MC often results in a reduction in MS volume on the affected side, compared to the non-impacted side.^4^ This reduction may be attributed to the altered bone structure and the pressure exerted by the impacted tooth on the surrounding tissues. Furthermore, a study by Alhaija et al.^5^ evaluated MS volume in patients with unilaterally buccally and palatally impacted MCs, and found that the affected side exhibited a significantly smaller MS volume in palatally impacted MCs and increased MS volume in buccally impacted MCs. This was due to closeness of roots of palatally impacted MCs to MS. These studies highlighted the impact of dental anomalies on adjacent anatomical structures and reinforced the need for a detailed radiographic evaluation before initiating orthodontic treatment.

Following orthodontic intervention to guide the impacted MC into proper alignment, a study has shown a tendency for the MS volume to increase, eventually resembling the volume of the unaffected side,^4^ suggesting that MS morphology can adapt dynamically in response to orthodontic treatment. This change can be attributed to the bone remodelling process that occurs as the MC erupt into the arch, relieving pressure on the MS walls and allowing for a more normalized MS cavity. Nevertheless, the researchers executed the investigation utilizing a limited sample size, exhibiting statistical power below 80%, and they did not evaluate sex-based differences in MS volumes, as well as the positioning of MC roots in relation to the floor of the MS was not evaluated. Furthermore, they failed to distinguish whether the MCs were impacted buccally or palatally. These represent the confounding variables that may potentially influence the findings and warrant further examination. Contradictory results are available in literature regarding sex-based differences in MS volumes. Nunes Rocha et al.^6^ found greater MS volume in males, which was in contrast with the study by Alhaija et al.^5^ who reported non-significant sex differences. 

The dynamic nature of MS volume adaptation following orthodontic disimpaction suggests that, while impacted MC may contribute to transient sinus alterations, these changes are reversible with appropriate treatment. This reinforces the importance of precise diagnosis and individualized treatment planning to achieve not only optimal occlusion, but also harmonious anatomical relationships between dental and MS structures. Due to lack of studies in this area, this study aimed to evaluate MS volume in cases of unilaterally displaced MCs (buccal and palatal) compared to the non-displaced side, and assess MS volume changes following orthodontic treatment. The primary objective was to compare MS volume variations based on sex and impaction type. Secondary objectives included identifying potential predictors of MS volume changes, such as age, sex, impaction type, treatment duration, side of impaction, and proximity to the MS floor.

## MATERIAL AND METHODS

### STUDY DESIGN AND SETTING

This prospective study was conducted using CBCT scans of patients with unilaterally impacted MCs who underwent orthodontic treatment at Department of Orthodontics between September 2020 to June 2024. Ethical approval was obtained from the institutional review board. CBCT scans were retrieved from the radiology database, and written informed consent was obtained as a routine protocol of the department from all the patients, before starting the study, to use their records for study purpose. This study adhered to the ethical guidelines of the Declaration of Helsinki. Patient confidentiality was maintained by anonymizing CBCT data. 

### PATIENTS’ ELIGIBILITY

Patients older than 16 years, irrespective of sex, with skeletal Class I pattern (ANB = 2-4°), who had unilaterally displaced MCs, fully erupted permanent dentition up to the second molar, except the presence of unilateral impacted MCs, no history of maxillofacial trauma, MS pathology, previous surgical interventions in the maxillary region, breathing abnormalities, and those who had sufficient space available for alignment of impacted MCs, were selected for the study. Patients with presence of congenital anomalies or syndromic conditions affecting craniofacial structures, history of previous orthodontic treatment, bilaterally impacted MCs, and patients with a history of MS surgery or chronic sinusitis were excluded.

### SAMPLE SIZE ESTIMATION

The sample size estimation was performed using G*Power software (version 3.2.9, Heinrich-Heine-Universität Düsseldorf, Germany) to achieve a statistical power of 80%, with a 5% significance level (alpha error). Based on a minimum effect size of 0.64, derived from a previous study by Oz et al.^4^, a total of 60 teeth (30 per group) were determined to be sufficient. The referenced study assessed MS volume in patients with impacted MCs, and compared it with healthy controls. Given that the present study followed a split-mouth design, a sample size of 30 patients was considered adequate. Considering 10% loss to follow-up, the present study was conducted on 33 patients.

### METHODOLOGY

A non-extraction treatment was done for all the patients with 0.022 x 0.028-in preadjusted brackets (3M, Minnesota, USA). The impacted MCs were exposed using a closed technique, and the orthodontic attachments were bonded to apply light forces to bring impacted MCs into alignment. Initially, a K-9 spring was used to direct forces appropriately for MC eruption, and once the MC emerged into the oral cavity, elastomeric thread was utilized to continue controlled traction for most of the cases. However, in some cases alternate mechanics were applied based on case requirement. The average orthodontic treatment time was 20.04 ± 5.13 months. All surgical procedures for disimpaction of MCs and orthodontic treatment were performed by the same oral surgeon and an orthodontist, respectively, with more than eight years of clinical experience, to eliminate bias due to inter-operator variability. 

CBCT scans were obtained using a i-CAT (Imaging Sciences Int. Inc., Pennsylvania, USA) with the following parameters: field of view (FOV) = 6 x 8-cm, voxel size = 0.3 mm, tube voltage = 90 kVp, and tube current = 5 mA. All scans were taken with patients in a standard head position, to ensure consistency. The axial section was calibrated to align with the Frankfort horizontal plane, the sagittal section was calibrated to correspond with the midsagittal plane, and the coronal section was calibrated to traverse the furcation of the maxillary first molar roots.

The images were captured in the digital imaging and communications in medicine (DICOM) format, wherein each DICOM file encapsulates a single frame with a resolution of 512 x 512 matrix. MS volume was assessed following a semi-automatic segmentation protocol, using ITK-SNAP software (version 3.8, ITK-SNAP, UPenn & UNC, USA). Two demarcating lines were used to delineate the panoramic section: a vertical line (V) and a horizontal line (H) situated at the center of the dental arch. The horizontal line was positioned at the utmost mesiodistal extension of the sinus cavity; conversely, the vertical line was ascertained from the lowest point of the sinus floor to its apex, which aligned with the boundary of the orbital floor. It extended specifically from the medial wall of the nasal cavity to the distal wall adjacent to the tuberosity of the maxilla. 

**Figure 1:**
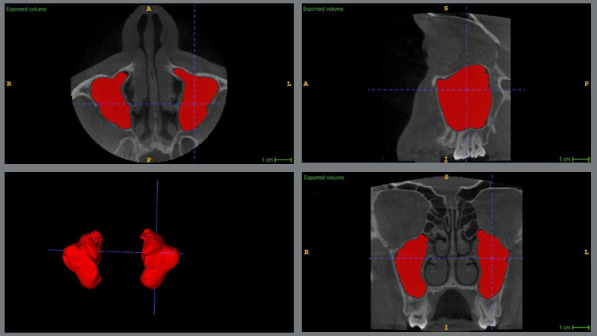
Volumetric assessment of maxillary sinus volume using semi-automatic segmentation method.

ITK-SNAP software uses an active contour segmentation algorithm to facilitate precise and reproducible volumetric analysis. The process began with the manual placement of seed points within the MS cavity on axial, sagittal, and coronal CBCT slices, to initialize the region of interest. The software then automatically expanded these seeds to delineate the sinus boundaries, based on Hounsfield Unit (HU) thresholds, set to distinguish bone (HU > 700) and soft tissue interfaces. Manual adjustments were made to refine the segmentation, particularly at complex anatomical boundaries, such as the sinus floor or osteomeatal complex, to ensure accuracy. To determine MS volume, “bubbles” and spicules were removed from the original segmentation, leaving the final three-dimensional model with a smoother surface. The ITK-SNAP methodology was chosen due to its validated reproducibility in neuroimaging applications^7^^,^^8^ and its ability to efficiently handle large CBCT datasets, while maintaining high precision. The final volumetric assessment of MS was performed and measured in mm^3^, independently by two calibrated examiners, to minimize interobserver variability. Any discrepancies greater than 5% were reassessed and resolved through consensus (Fig. 1). The vertical relationship between MS floor and root apices of impacted MCs was measured and classified according to Khojastepour et al.^9^

## RELIABILITY ASSESSMENT

To assess intra- and inter-observer reliability, ten CBCT scans were randomly selected and remeasured after two weeks. Intraclass correlation coefficients (ICC) were calculated, with values > 0.80 considered indicative of excellent reliability.

## STATISTICAL ANALYSIS

Statistical analysis was performed using SPSS software (IBM Corp., 2013, IBM SPSS Statistics for Windows, version 23.0, Armonk, NY, USA). Data normality was evaluated using the Shapiro-Wilk test and further verified through a Q-Q plot, confirming a normal distribution. Categorical variables were expressed as frequency and percentage, and their distribution across impacted MC position, location, and MS normalization was analyzed using the chi-square test. Continuous variables, such as age, treatment time, and MS volume, were reported as mean and standard deviation (SD). Group comparisons for mean values were conducted using an independent t-test. Additionally, a multifactorial regression analysis was performed to identify predictor variables influencing sinus volume normalization. Statistical significance was set at p < 0.05, with Bonferroni correction applied to adjust for multiple comparisons and reduce the risk of Type I errors.

## RESULTS

The inter-observer ICC values for reliability testing was 0.89 and for intra-observer, it was 0.91, which showed excellent reliability and reproducibility. Out of 33 patients, three patients were lost to follow-up as they did not complete their treatment. A per-protocol analysis plan was chosen for this study, as it excludes protocol violators (such as non-compliant patients), and avoids dilution of the treatment effect caused by non-compliance. A comparative analysis of baseline characteristics between the sexes was conducted using the chi-square test. The sample consisted of 18 (60%) buccally placed MCs, and 12 (40%) palatally impacted MCs, which showed that the distribution of buccally impacted MCs was higher than palatally impacted MCs. The type of vertical relationship between the root apices of MC and MS floor was Type II in 18 (60%) patients, and Type I in 12 (40%), patients. The MS volume achieved normalcy in 14 (46.7%) patients. The chi-square statistic for MC position indicated no statistically significant association between sex and MC position, location, and in achieving MS volume normalization (Table 1). These findings suggested that these variables were independent of sex within the study population.

**Table 1:** Comparison of baseline characteristics of study population.

Parameters	Category	Male		Female		Total	Chi-stats	p-value
		n	%	n	%			
Impacted canine position	Buccal	10	33.3%	8	26.7%	18 (60.0%)	0.37	0.543
	Palatal	8	26.7%	4	13.3%	12 (40.0%)		
Impacted canine location	Type I	6	20.0%	6	20.0%	12 (40.0%)	0.83	0.361
	Type II	12	40.0%	6	20.0%	18 (60.0%)		
Sinus volume achieved normalcy	No	10	33.3%	6	20.0%	16 (53.3%)	0.09	0.765
	Yes	8	26.7%	6	20.0%	14 (46.7%)		

p-value > 0.05: Non-significant. Data is presented in form of n (%).

The comparison of baseline characteristics between the patients was analyzed using an independent t-test. The mean age of male patients was 21.56 ± 1.89 years, while that of female patients was 22.5 ± 1.78 years. No statistically significant difference in age was found between the two groups (p = 0.181). Similarly, the mean treatment time for males was 21.78 ± 2.26 months, whereas for females, it was 20.17 ± 2.98 months, with no statistically significant difference (p = 0.104). This suggests that sex-based differences did not play a crucial role in these baseline characteristics within this sample (Table 2).

**Table 2:** Comparison of baseline characteristics of study population.

Parameter	Sex	n	Mean	SD	t-stats	p-value
Age in years	Male	18	21.56	1.886	-1.37	0.181
	Female	12	22.5	1.784		
Treatment time in months	Male	18	21.78	2.264	1.68	0.104
	Female	12	20.17	2.98		

p-value > 0.05: Non-significant. Data is presented in form of mean and standard deviation (SD).

For pretreatment MS volume, there was no significant difference between males and females (p = 0.75) or right and left impacted sides (p = 0.468). However, a statistically significant difference was observed between buccal and palatal MC positions (p = 0.002), where increased MS volume was noticed with buccally impacted MCs. Post-treatment MS volume showed no significant differences across sex (p = 0.3), impacted side (p = 0.236), or position (p = 0.597). The mean change in MS volume was greater with palatally impacted MCs (p = 0.044), which showed a significant increase in the MS volume, compared to buccally impacted MCs. The distance of the MC root tip from the MS floor was significantly greater in females, suggesting a higher placement of MCs in males, compared to females. The MS volume required to achieve normalcy was not significantly different across sex (p = 0.406), impacted side (p = 0.351), or position (p = 0.509). However, the right and left MS volume difference (pretreatment) was significantly different based on MC position (p = 0.035), with increased MS volume for palatally impacted MCs (Table 3). These results suggested that buccal and palatal positions of impacted MC may have a more pronounced effect on MS volume changes, compared to sex or impacted side.

**Table 3:** Comparison of outcome variables of study population with impacted canine.

Parameters	Sex^†^		Impacted canine side^‡^		Impacted canine position^‡^	
	Male	Female	Right	Left	Buccal	Palatal
Maxillary sinus volume (Pretreatment) in mm^3^	10822.44 ± 871.88	11193 ± 667.3	11128.88 ± 758.37	10789.86 ± 846.94	11356.89 ± 560.71	10391.33 ± 783.51
	0.75		0.468		0.002*	
Maxillary sinus volume (Post-treatment) in mm^3^	12888.89 ± 821.45	12593.83 ± 803.89	12941.25 ± 860.03	12576.14 ± 739.88	12873 ± 635.41	12617.67 ± 1038.94
	0.3		0.236		0.597	
Mean change in maxillary sinus volume in mm^3^	1928.11 ± 875.58	1250 ± 628.62	1677.38 ± 932.12	1633.43 ± 766.78	1382.78 ± 785.68	2068 ± 787.16
	0.244		0.522		0.044*	
Distance of canine root tip from sinus floor (mm)	2.33 ± 0.49	3.5 ± 1.17	3 ± 1.15	2.57 ± 0.76	2.67 ± 1.08	3 ± 0.85
	0.006*		0.247		0.202	
Volume required to achieve normalcy in mm^3^	-276.67 ± 69.79	-301.67 ± 141.09	-270 ± 93.81	-305.71 ± 112.57	-266.67 ± 93.43	-316.67 ± 112.76
	0.406		0.351		0.509	
Right and left sinus volume difference (Pretreatment) in mm^3^	1990 ± 853.17	1516.67 ± 506.02	1887.5 ± 792.36	1701.43 ± 740.49	1556.67 ± 705.37	2166.67 ± 717.74
	0.258		0.514		0.035*	

*p<0.05: significant, ^†^Mann Whitney U test, ^‡^ Independent t test.

**Figure 2:**
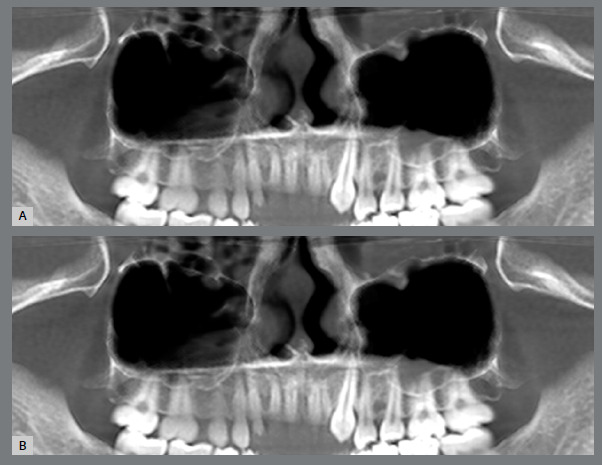
Maxillary sinus volume before **(A)** and after orthodontic alignment **(B)** of unilaterally impacted maxillary canine.

The pretreatment MS volume was significantly lower on the impacted side (10970.67 ± 805.37 mm^3^), compared to the non-impacted side (12698.00 ± 791.86 mm^3^) (p < 0.001). However, after treatment, the post-treatment MS volume showed no statistically significant difference between the impacted (12770.87 ± 813.82 mm^3^) and non-impacted sides (12914.20 ± 799.29 mm^3^) (p = 0.468). The mean change in MS volume difference was significantly greater on the impacted side (1656.87 ± 844.67 mm^3^), compared to the non-impacted (216.20 ± 70.16 mm^3^) side (p < 0.001), suggesting that the impacted side experienced a larger volumetric change following treatment. These results indicated that the greater MS volume difference on the impacted side highlights the extent of MS adaptation following intervention (Fig. 2).

A multivariate regression analysis was performed to identify the predictor variables influencing MS volume normalization during treatment. The analysis revealed that age (p = 0.004), sex (female) (p = 0.003), impacted MC position (palatal) (p = 0.002), treatment time (p = 0.001), right-left MS volume difference (pretreatment) (p = 0.037), and distance of the MC root tip from the MS floor (p = 0.017) were statistically significant predictors. Conversely, impacted MC side (p = 0.500) was not a significant factor in determining MS volume normalization. The findings indicated that younger age, palatally impacted MCs, and greater pretreatment side-based MS volume difference were significantly associated with achieving near-normal MS volume with orthodontic treatment of impacted MCs. Additionally, longer treatment time was negatively correlated with MS volume normalization, suggesting that prolonged treatment might hinder optimal MS volume recovery. Farther the root tip of MC from the MS floor, better are the chances to achieve normalcy. However, the side of impaction did not significantly impact the outcome, implying that laterality did not contribute meaningfully to MS volume changes during treatment (Table 4).

**Table 4:** Multivariate regression analysis for predictor variables influencing sinus volume normalization.

Parameters	Unstandardized	Standard Error	t value	p-value
Age	20.185	6.229	3.24	0.004*
Sex (Female)	-114.025	34.045	-3.34	0.003*
Impacted canine side (Right)	19.168	27.955	0.68	0.500
Impacted canine position (Palatal)	141.777	39.891	3.55	0.002*
Treatment time	-47.414	7.712	-6.14	0.001*
Right and left sinus volume difference (Pretreatment)	0.037	0.017	2.21	0.037*
Distance of canine root tip from sinus floor	-15.348	13.644	2.56	0.017*

*p-value < 0.05: significant.

## DISCUSSION

The present study aimed to assess the volumetric changes in the volume of MS after orthodontic treatment of impacted MC, and to assess the impact of various factors on MS volume changes in patients. The findings highlight the complex interplay between sex, MC position, impacted side, and treatment time in influencing MS volume normalization. Only patients exhibiting unilateral MC impaction were incorporated into the study, to avoid the influence of any confounding variables. All participants included in the research were aged 16 years or older; therefore, we did not anticipate any alterations in MS volume during the fixed orthodontic intervention that could be attributed to the growth of the patients. 

The use of ITK-SNAP for semi-automatic delineation of the MS volume in this study offered distinct advantages over traditional manual segmentation methods, which rely on labour-intensive hand-tracing of sinus boundaries across multiple CBCT slices. ITK-SNAP’s active contour algorithm, guided by initial seed points and HU thresholds, enhanced efficiency and reduced subjective variability, as evidenced by the high inter-observer (ICC = 0.84) and intra-observer (ICC = 0.89) reliability in our results. Pinheiro et al.^10^ conducted a comparative analysis of ITK-SNAP and Dolphin 3D software regarding their accuracy in three-dimensional volumetric evaluation of the pharyngeal airway, revealing inter- and intra-examiner ICC values ranging from 0.91 to 1.00, thereby indicating a high degree of reliability for both software applications. Compared to manual methods, which are time-consuming and prone to inter-operator inconsistencies, semi-automatic segmentation with ITK-SNAP provides a standardized and reproducible workflow, as supported by prior neuroimaging studies.^7^^,^^8^

As articulated by El and Palomo^11^, the method of automatic segmentation, frequently employed for quantifying airway volume, is straightforward; however, it is deficient in precision. They propose that segmentation should be executed in a semi-automatic manner, which entail the integration of both automatic and manual segmentation techniques, thereby enhancing the accuracy of the measurements. However, the semi-automatic approach still requires manual refinement at complex anatomical interfaces, which may introduce minor variability, particularly in cases with irregular sinus morphology. While manual segmentation allows for greater operator control, it is less practical for large datasets and may compromise reproducibility. Future studies could explore fully automated segmentation techniques to further minimize subjectivity, while comparing their accuracy against ITK-SNAP’s semi-automatic approach, to establish a gold standard for MS volumetric analysis.

The mean change in the volume of the MS was observed on both the impacted and non-impacted sides, albeit the change on the non-impacted side was deemed to be negligible. This observation indicates that orthodontic intervention induces an augmentation in the volume of the MS, a finding that is corroborated by prior research.^12^^,^^13^ This could be due to the fact that during the process of orthodontic displacement of the tooth in conjunction with the MS, the migrating root is relocated into the alveolar bone through the mechanisms of surrounding bone resorption and apposition. The systems of alveolar bone modelling and remodelling exhibit a remarkable capacity to adapt swiftly to alterations in mechanical loading. The induction of new bone formation on the floor of the MS can be facilitated by the movement of orthodontically repositioned teeth.^14^

The results of the study indicated that MS volume was significantly greater with buccally impacted MCs, compared to palatally impacted MCs. This finding was in agreement with the study by Alhaija et al.^5^ Moreover, palatally impacted MCs exhibited a higher degree of MS volume increase following orthodontic intervention. The greater volumetric change in palatally impacted cases could be attributed to the relative proximity of the impacted tooth to the MS floor. The mechanical forces applied during orthodontic movement likely contribute to MS adaptation, with more pronounced remodelling occurring in palatal impactions, due to their deeper placement within the alveolar bone. These findings underscore the need for tailored treatment strategies based on MC position, as palatally impacted MCs may require more careful monitoring to ensure optimal MS adaptation. This finding could not be compared due to lack of study on this topic. Oz et al.^4^ ascertained that the orthodontic intervention for impacted MC markedly augmented the volume of the MS; nevertheless, they did not evaluate whether this augmentation resulted in the normalization of the volume. 

It was indicated by the present study that younger age, palatally impacted MCs, shorter treatment duration, increased distance of the root tip of impacted MC from MS floor, and pretreatment MS volume differences were significant predictors of MS volume normalization. The prolonged treatment duration could be associated with prolonged inflammatory response, and the chronic mechanical stress can lead to sustained bone resorption and remodelling, which may delay MS volume stabilization. Kuroda et al.^15^ undertook an investigation involving rats, wherein they observed the influence of orthodontic forces on alterations in bone remodelling at the floor of the MS, leading to the conclusion that the mechanotransduction of mechanical stress exerted on a tooth over a duration of two weeks induced bone formation on the surface of the MS. Kopecka et al.^16^ suggested that the more MS pneumatization occurred in the cases where the root tip of the impacted MC was more than 2mm away from the MS floor. Younger individuals have a higher rate of bone turnover and remodelling, which allows for faster structural adaptation. This helps the MS cavity adjust to anatomical and functional demands more efficiently than in older individuals.^17^

The prior research has indicated that the measurements of the MS are more pronounced in males than in females^6^, a phenomenon attributed to the elevated functional requirements in males, which can be ascribed to their larger body mass and more extensive craniofacial skeleton. Conversely, in the present investigation, both male and female participants exhibited negligible discrepancies in the volume of the MS. This finding aligns with the observations made by Urooge and Patil^18^, and Alhaija et al.^5^, who noted a statistically non-significant sex variation concerning the MS length, height, area, and volume. It is also plausible that the limited representation of males and females in the current research may have obscured any potential sex-related distinctions.

The results of the present study further indicated that MS volume was significantly greater for palatally impacted MCs on the right side. This might be due to the fact that root tip of the palatally impacted MCs was away from MS floor. The MS expands through a process called pneumatization, which occurs during growth. If the impacted MC’s root tip is farther from the MS floor, there is less interference with the MS’s natural expansion.^9^ This could allow the MS to develop to a larger volume, compared to cases where the MC is closer to or impinging on the MS floor. An impacted MC that is close to the MS floor may exert pressure on it, restricting its downward expansion.^4^

### CLINICAL IMPLICATIONS

The clinical implications of these findings highlight the importance of individualized treatment planning for impacted MC based on their position and proximity to the MS. Since palatally impacted MCs exhibited greater MS volume changes post-treatment, careful monitoring is required to ensure proper MS adaptation and prevent complications such as MS membrane perforation or delayed bone remodelling. Additionally, orthodontists should consider that a greater distance between the MC root tip and the MS floor facilitates MS pneumatization, potentially improving treatment outcomes. Shorter treatment durations may enhance MS normalization, emphasizing the need for efficient mechanics to minimize prolonged mechanical stress and inflammatory responses. CBCT should be incorporated into diagnostic protocols to assess MS volume and anatomical variations before treatment. Understanding these factors can help optimize orthodontic strategies, ensuring successful eruption guidance, while minimizing sinus-related complications and structural imbalances during orthodontic interventions for impacted MC.

## LIMITATIONS

Despite the valuable insights provided, this study has certain limitations. First, the sample size, though statistically justified, may not fully represent broader populations with varying anatomical and developmental characteristics. Second, CBCT scans were obtained from a single institution, potentially affecting external validity. Additionally, the study did not account for genetic or environmental factors influencing MS volume. The segmentation process, despite using semi-automated methods, may still involve subjective elements, introducing measurement variability. Furthermore, treatment mechanics varied slightly among patients, which might have influenced MS adaptation differently. Lastly, the absence of long-term follow-up data prevents an assessment of post-treatment stability and potential relapse in MS volume changes. Future prospective studies with larger, diverse samples and longitudinal follow-up are needed to validate and extend these findings.

## CONCLUSION

Within the scope of this study, the following conclusions were drawn:

1. This study demonstrated that orthodontic treatment of unilaterally impacted MCs led to significant volumetric changes in the MS, with greater adaptation observed on the impacted side.
2. The MS volume was higher on non-impacted side, and it was significantly greater with buccally impacted MCs. Palatally impacted MCs exhibited a greater increase in MS volume post-treatment, compared to buccally impacted MCs.
3. Factors significantly associated with MS volume normalization included: younger age, palatal impaction, shorter treatment duration, increased pretreatment MS volume difference, and greater distance of the MC root tip from the MS floor.
4. While sex and impacted side did not significantly influence post-treatment MS volume, prolonged treatment duration was negatively correlated with MS adaptation.

## Data Availability

All data generated or analyzed during this study are included in this published article.
